# Intracellular Spread of Rabies Virus Is Reduced in the Paralytic Form of Canine Rabies Compared to the Furious Form

**DOI:** 10.1371/journal.pntd.0004748

**Published:** 2016-06-02

**Authors:** Shanop Shuangshoti, Paul Scott Thorner, Chinachote Teerapakpinyo, Nisachol Thepa, Pornchai Phukpattaranont, Nirun Intarut, Boonlert Lumlertdacha, Veera Tepsumethanon, Thiravat Hemachudha

**Affiliations:** 1 Department of Pathology, Faculty of Medicine, Chulalongkorn University, Bangkok, Thailand; 2 Chulalongkorn GenePRO Center, Research Affairs, Faculty of Medicine, Chulalongkorn University, Bangkok, Thailand; 3 WHO Collaborating Center for Research and Training on Viral Zoonoses, Bangkok, Thailand; 4 Department of Laboratory Medicine, Hospital for Sick Children and University of Toronto, Toronto, Canada; 5 Department of Clinical Pathology, Faculty of Medicine, Chulalongkorn University, Bangkok, Thailand; 6 Department of Electrical Engineering, Faculty of Engineering, Prince of Songkla University, Songkhla, Thailand; 7 Epidemiology Unit, Faculty of Medicine, Prince of Songkla University, Hat Yai, Songkhla, Thailand; 8 Queen Saovabha Memorial Institute, Bangkok, Thailand; 9 Department of Medicine, Faculty of Medicine, Chulalongkorn University, Bangkok, Thailand; Florida Gulf Coast University, UNITED STATES

## Abstract

Studies of the furious and paralytic forms of canine rabies at the early stage of disease have shown a more rapid viral colonization of the cerebral hemispheres in the furious form, as measured by viral antigen within neuronal cell bodies and viral RNA levels. Measurement of cellular processes separate from neuronal cell body provides a visual record of the spread of rabies virus which occurs across synapses. In this study, the amount of rabies viral antigen within cell processes was quantitatively assessed by image analysis in a cohort of naturally rabies infected non-vaccinated dogs (5 furious and 5 paralytic) that were sacrificed shortly after developing illness. Measurements were taken at different levels of the spinal cord, brain stem, and cerebrum. Results were compared to the amount of rabies viral antigen in neuronal cell bodies. Generally, the amount of rabies viral antigen in cell processes decreased in a rostral direction, following the pattern for the amount of rabies viral antigen in neuronal cell bodies and the percentage of involved cell bodies. However, there was a delay in cell process involvement following cell body involvement, consistent with replication occurring in the cell body region and subsequent transport out to cell processes. Greater amounts of antigen were seen in cell processes in dogs with the furious compared to paralytic form, at all anatomic levels examined. This difference was even evident when comparing (1) neurons with similar amounts of antigen, (2) similar percentages of involved neurons, and (3) anatomic levels that showed 100% positive neurons. These findings suggest that intracellular transport of the virus may be slower in the paralytic form, resulting in slower viral propagation. Possible mechanisms might involve host-specific differences in intracellular virus transport. The latter could be cytokine-mediated, since previous studies have documented greater inflammation in the paralytic form.

## Author Summary

Dogs with rabies can show the furious or paralytic form. The virus spreads from nerve cell to nerve cell via connections in cell processes. There are greater amounts of virus in the nerve cell bodies in the furious form. Studying cell processes separate from cell body provides a visual record of the spread of rabies virus. The amount of rabies viral protein within cell processes was measured in dogs with rabies (5 furious and 5 paralytic) sacrificed shortly after developing illness. The amount of viral protein in cell processes decreased from spinal cord to brain, as did the amount of viral protein in cell bodies and the percentage of involved cell bodies. However, there was a delay in cell process involvement following cell body involvement, consistent with the virus replicating in the cell body region and later moving out to cell processes. Greater amounts of viral protein were seen in cell processes in dogs with the furious compared to paralytic form, by comparing nerve cells with similar amounts of antigen, or similar percentages of involved nerve cells. These findings suggest that intracellular transport of the virus may be slower in paralytic rabies, resulting in slower viral spread in the brain.

## Introduction

Rabies is an almost uniformly fatal infectious disease of the central nervous system (CNS), caused by a neurotropic RNA virus in the family *Rhabdoviridae*, genus *Lyssavirus* [1]. The worldwide number of rabies deaths was estimated to be 70,000 in 2011 [2]. Furious and paralytic clinical forms of rabies occur in both humans and dogs in a ratio of approximately 2:1 [3]. While limbic symptoms dominate the clinical picture in the furious form, paralysis of lower motor neuron type is the major clinical feature of the paralytic form [3, 4].

Differences between these two forms of rabies cannot easily be explained by postmortem histopathological studies. Rabies viral nucleocapsid antigen was found to be confined mainly to midline structures of the CNS such as thalamus, basal ganglia, and brainstem in human cases at autopsy, and inflammation was generally mild throughout the neuraxis [5]. A higher RNA viral load with less immune response has been reported in the brain in the furious form [6]. Spinal cord anterior horn cell dysfunction with central chromatolysis of the neurons at corresponding bite level (but without clinical weakness) has been demonstrated in the furious form of rabies while dysfunction of peripheral nerves, axon- or myelinopathy has been found in the paralytic form [3, 4]. One hypothesis is that there is selective functional impairment rather than selective tissue involvement, in order to explain, for example, the limbic aggression in furious rabies [3]. Functional alteration of muscarinic acetylcholine receptors has been observed in rabid dog brains, especially in the brainstem and hippocampus [7].

Examination of animals and humans late in the course of the disease may obscure the differences between the two clinical forms [2]. Our previous studies in naturally infected dogs during the early disease stage showed disruption of axonal integrity in the brainstem exclusively in paralytic dogs from pronounced inflammation [2, 8–9]. Brainstem inflammation was postulated to impede viral propagation to the hemispheres, resulting in a reduced viral burden in the paralytic dog brains [2]. We also previously demonstrated that the percentage of neuronal cell bodies containing rabies viral nucleocapsid antigen decreased in a caudal-rostral direction [2]. This pattern was the inverse of the level of rabies viral RNA, with higher levels in the cerebrum than in the brainstem and spinal cord. The viral RNA likely reflects an earlier stage of infection with detectable viral protein occurring at a later stage. However, the higher RNA levels in furious rabies raise the possibility that a slower rate of viral propagation might be occurring in the paralytic form. Although rabies viral antigen has been previously demonstrated beyond the neuronal cell body (in neuronal processes and neuropil) [10], quantitative measurements have not been performed. Such measurements provide a visual record of intracellular spread. The present study aimed to compare intracellular viral spread (and thereby viral propagation) between paralytic and furious forms of rabies, by determining the amount of rabies viral antigen beyond the neuronal cell body at different CNS levels.

## Methods

### Specimens

Animals in this study were the same set as previously described [2]. Briefly, ten naturally rabies-infected dogs were available from the rabies diagnostic unit of the Queen Saovabha Memorial Institute, Bangkok, Thailand. All dogs were in the early stage of infection, including five with the furious form and five with the paralytic form. Dogs had no abnormal signs when first brought in, and underwent euthanasia within 36 hours of developing the first clinical sign. Previous observations made on 957 confirmed rabid dogs quarantined until death, had determined that the median survival time of rabid dogs was 4 days, with 25% dying within 48 hours, and usually lapsing into coma 12 hours before death [2]. On this basis, all animals enrolled in the present study were considered to be in the early stage of disease.

Brains were available in all cases and spinal cords from six cases (three of each clinical form). A canine brain transaction atlas [11] was used to identify the region of interest as follows: frontal tip, parietal lobe (level 2), temporal lobe (level 5), occipital tip, cerebellum (vermis), caudate nucleus (level 2), thalamus (level 4), hippocampus (including CA1–4 regions) (level 5), midbrain (level 7), pons (level 12), medulla (level 17), and spinal cord (cervical, thoracic, lumbar, and sacral levels). One section was taken from each brain and brainstem region and 3 from each spinal cord level. All specimens were fixed in 10% formalin, processed and embedded in paraffin wax. Four-micron-thick sections were stained with hematoxylin and eosin. Immunohistochemical staining was performed using an automated stainer (Ventana Benchmark LT, Tucson, USA), and a polyclonal anti-rabies nucleocapsid antibody (Bio-Rad; Marnes-la-Coquette, France) at a dilution of 1:80. Negative control was performed by omission of the primary antibody.

### Ethics statement

Our study was carried out on post-mortem CNS specimens from dogs with rabies. Dogs with rabies are handled by the Queen Saovabha Memorial Institute (QSMI), which is affiliated with Chulalongkorn University. QSMI is a WHO Collaborating Center for Rabies Research on Prevention and Prophylaxis, and is also the national diagnostic center for rabies in animals in our country. The CNS is routinely examined to confirm the diagnosis. The material used in the present study was tissue remaining. No live animal experimentation was performed. The study was approved by Committee on Animal Research and Ethics (project # F-RC-005), Faculty of Medicine, Ramathibodi Hospital, Mahidol University, Bangkok, Thailand.

### Quantitative analysis of rabies antigen beyond neuronal cell body and in hippocampal dentate fascia

**Fig 1:**
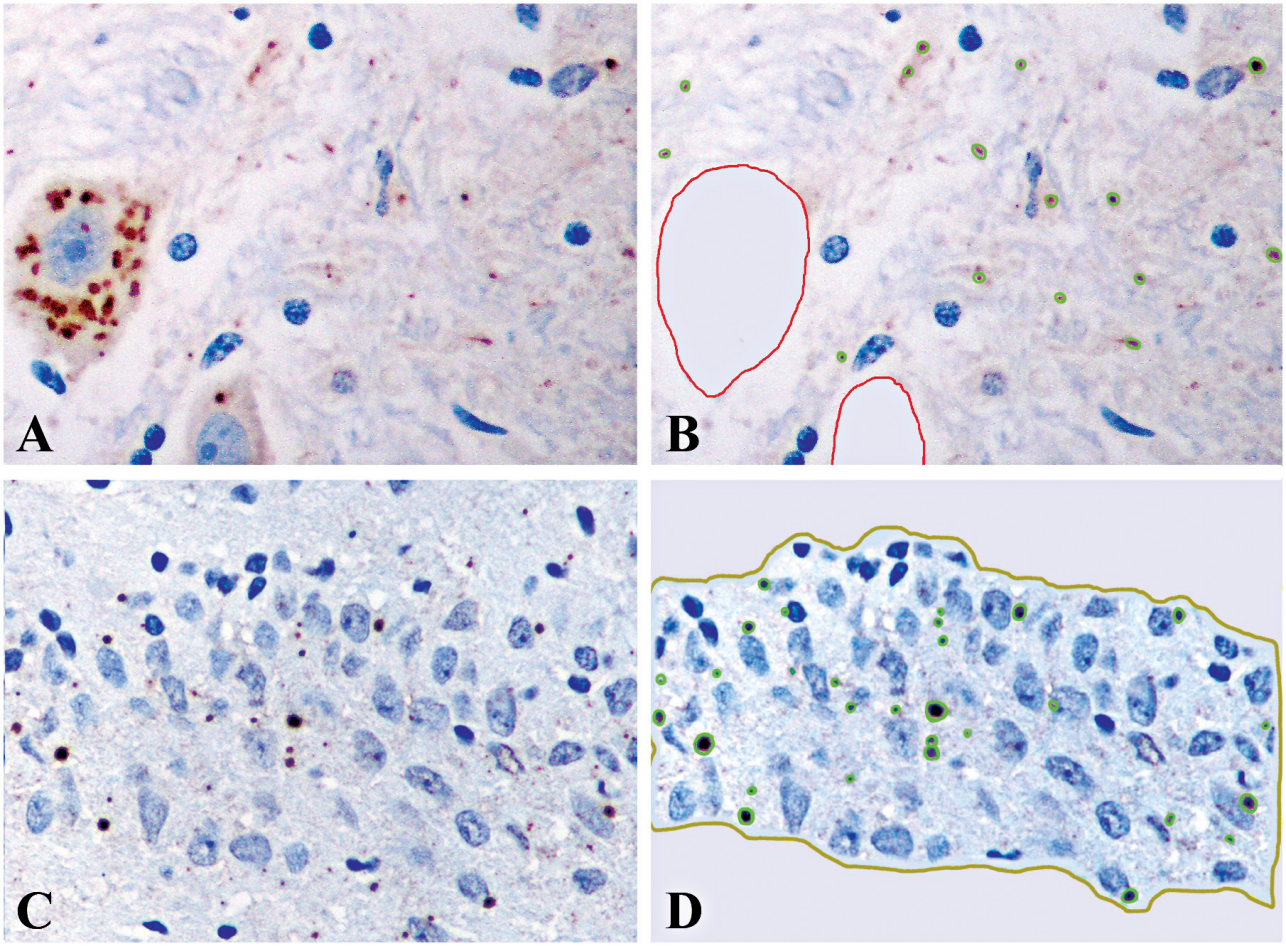
Image analysis for determination of rabies antigen within cellular processes (A and B) and the hippocampal dentate fascia (C and D). For all CNS regions (except the dentate fascia of hippocampus), following immunostaining for rabies antigen (A), all neuronal cell bodies were manually outlined. The antigen signals within the cell body were then deleted to allow only the antigen in cellular processes to be detected and quantified by computer software (B). For the hippocampal dentate fascia (C), detection of the antigen was done by computer software after the area had been manually outlined (D). (A-D, immunoperoxidase using anti-rabies nucleocapsid antibody).

All immunostained slides were captured in color by a digital camera (Nikon DXM1200F) attached to a microscope (Nikon ECLIPSE 80i) (Nikon Instech Co., Ltd., Japan) at 400x magnification, covering an area of 0.0705 mm2. Fields captured were selected as follows: for cerebral cortices, caudate nucleus and thalamus; the area with maximum RABV antigen in neurons was chosen, and all layers of the cerebral cortices were captured; consecutive fields of the CA1 through CA4 regions and dentate fascia of hippocampus, as well as the dentate fascia; the Purkinje cell layer of the cerebellum; all groups of neurons in the brainstem; and the gray matter of the spinal cord at all levels. Between 10–20 fields (0.7–1.41 mm2) were captured for each slide. Images were saved as 3840 x 3072 jpeg files. Quantitative assessment was performed by a computer-aided system for microscopic images (Cell Image Analyzer), using a similar approach to that in the previous study on neuronal cell bodies [2]. For all anatomical areas, all neuronal cell bodies were manually outlined, and the antigen within the cells digitally erased. Antigen area outside the cell bodies was then measured relative to neuropil area in order to determine the percentage of RABV antigen area outside neuronal cell bodies (Fig 1A and 1B). For the hippocampal dentate fascia, the percentage of antigen area was obtained by measuring the antigen area relative to the entire dentate area that had manually been outlined prior to digital analysis (Fig 1C and 1D). This region of the hippocampus was examined separately because limbic symptoms predominate in the furious form of rabies and it was not specifically assessed in the previous study [2].

### Statistical analysis

The extent of the rabies antigen burden at different major anatomical levels (spinal cord, brainstem, cerebellum, cerebral midline structures, and cerebrum) was compared between the furious and paralytic groups of rabid dogs using the Mann-Whitney U test for a one-tailed test. A one-sided test was used because the semiquantitative analyses showed a trend towards a greater antigen burden in furious rabies [2]. Statistical analyses were performed using Statistical Package for Social Science software (SPSS version 17.0, SPSS Inc., Chicago, IL, USA). Results were considered statistically significant when p<0.05.

Pearson’s correlation was used to determine the strength of the relationship between viral antigen parameters within cell bodies (percentage of positive neurons and antigen area per neuron) and viral antigen area beyond the neuronal cell body. A simple linear regression model was then performed to predict the value of the antigen area beyond the neuronal cell body as the viral antigen parameter within cell bodies changed. A p value of <0.05 was considered significant. Coefficient values (r) >0.5, between 0.5 and 0.3, and <0.3 indicated strong, medium, and weak correlations, respectively. Statistical analysis was performed using R version 3.2.2.

## Results

**Fig 2:**
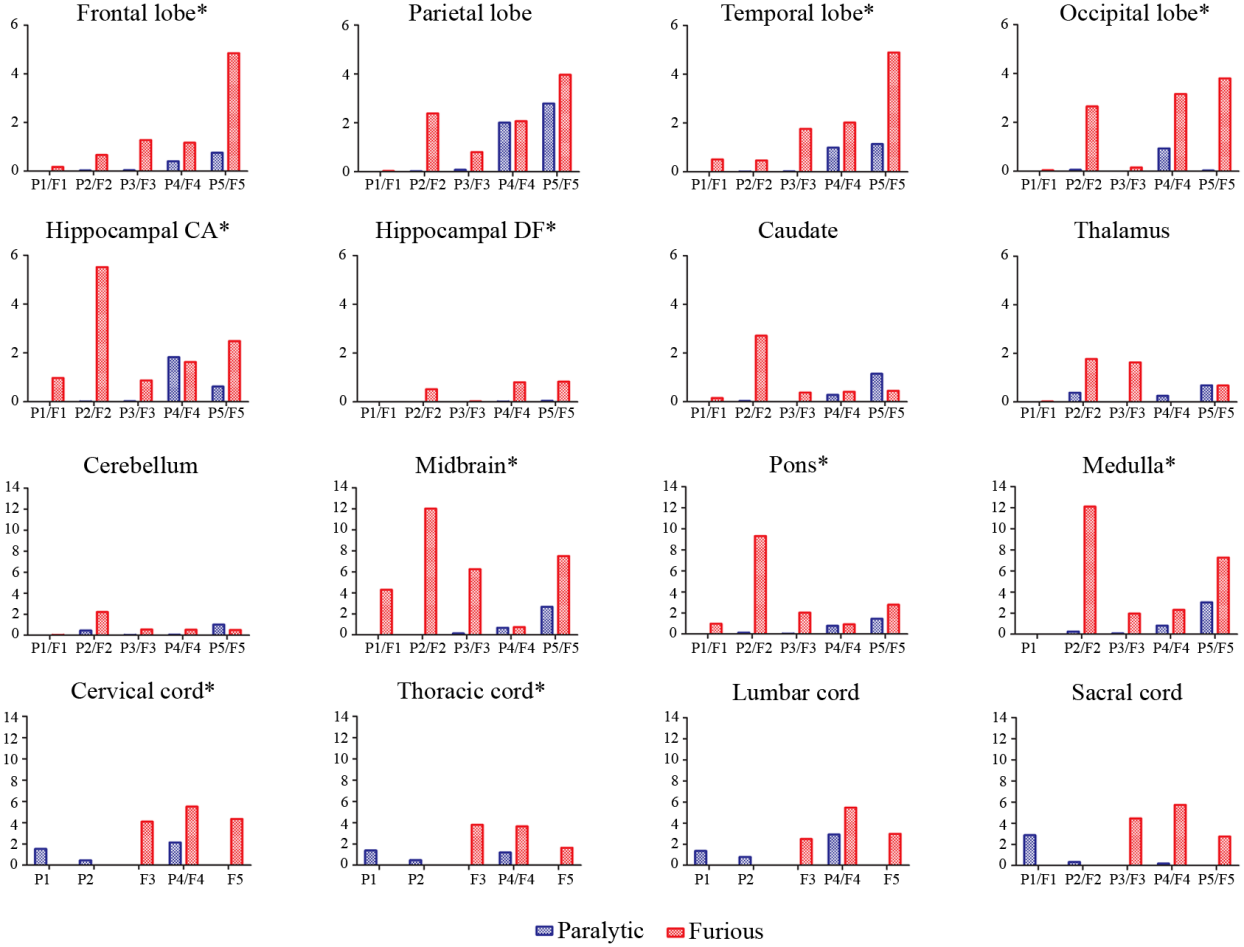
% RABA positive area outside the neuronal cell body is shown for individual dogs at each CNS regions. P1-P5 and F1-F5 in the X axis represent 5 paralytic and 5 furious canines, respectively. Spinal cord samples were not available in P3, P5, F1, and F2, while medulla oblongata was not available in F1. Values in the Y axis of all graphs are % RABA positive area outside the neuronal cell body. An asterisk following the label of a specific anatomical region indicates a significantly higher percentage of RABA area in furious as compared to paralytic dogs. CA = cornu ammonis; and DF = dentate fascia.

**Fig 3:**
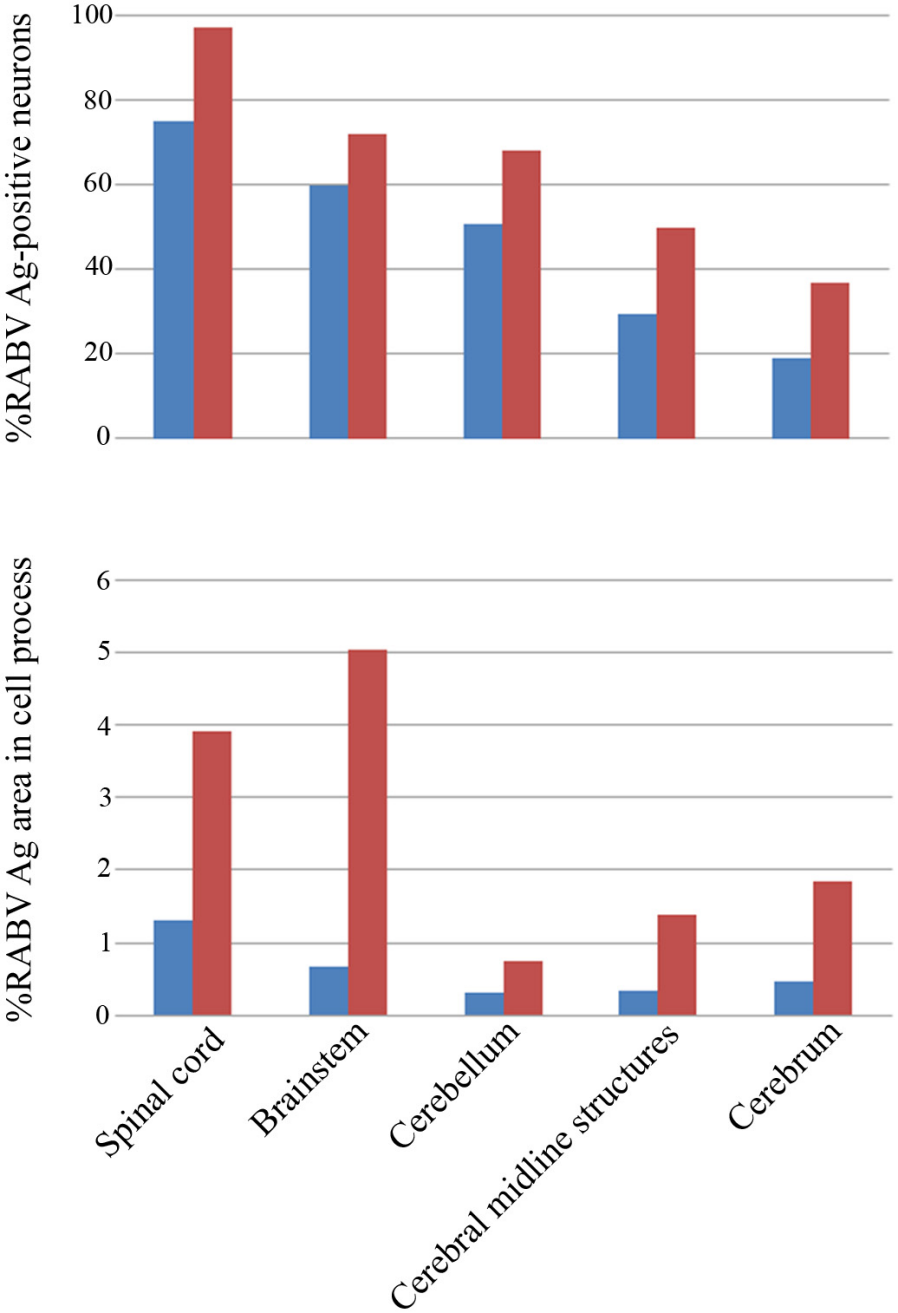
Bar graphs comparing dogs with furious rabies (red) and paralytic rabies (blue) with respect to percentage of neuronal cell bodies positive for rabies antigen (upper row), and percentage of areas in cellular processes positive for rabies antigen (lower row). Results are shown for each of five different levels of the CNS (spinal cord, brain stem, cerebellum, cerebral midline structures and cerebrum). The data on percentage of neuronal cell bodies positive for rabies antigen have been previously reported [2] and are shown here for comparison to the data on cellular processes.

Generally, rabies antigen beyond the neuronal cell body was decreased in a caudal to rostral direction for both furious and paralytic dogs. The value was most extensive at the brainstem and spinal cord levels of furious dogs. Results for individual dogs are presented in Fig 2. The percentage of the antigen-positive area beyond the neuronal cell body was greater in the furious as compared to paralytic dogs at all 16 levels examined, and reached statistical significance in 10 of these levels, including: frontal cortex, temporal cortex, occipital cortex, hippocampal cornu ammonis and hippocampal dentate fascia, brainstem (midbrain, pons, medulla) and in spinal cord (cervical and thoracic levels) (Table 1). The results are summarized in graphical form in Fig 3, and are displayed together with the previously published results on the neuronal cell body [2], in order to demonstrate the differences between the cell body and cell processes for the two clinical forms of rabies.

**Table 1:** Extent of rabies viral antigen beyond neuronal cell body.

	% RABV antigen-positive area (Mean ± SD)		
Locations	Paralytic	Furious	P value*
Frontal lobe	0.25 ± 0.33	1.63 ± 1.86	0.023
Parietal lobe	0.98 ± 1.33	1.85 ± 1.52	0.125
Temporal lobe	0.43 ± 0.58	1.93 ± 1.79	0.038
Occipital lobe	0.2 ± 0.41	1.96 ± 1.75	0.024
Hippocampal cornu ammonis	0.49 ± 0.79	2.29 ± 1.91	0.024
Hippocampal dentate fascia	0.01 ± 0.018	0.43 ± 0.4	0.042
Caudate	0.29 ± 0.49	0.82 ± 1.06	0.058
Thalamus	0.26 ± 0.28	1.02 ± 0.83	0.071
Cerebellum	0.3 ± 0.44	0.76 ± 0.84	0.087
Midbrain	0.69 ± 1.14	6.16 ± 4.15	0.008
Pons	0.47 ± 0.63	3.19 ± 3.49	0.014
Medulla	0.83 ± 1.26	5.9 ± 4.79	0.025
Cervical cord	1.38 ± 0.85	4.64 ± 0.75	0.025
Thoracic cord	1.01 ± 0.48	3.03 ± 1.2	0.025
Lumbar cord	1.69 ± 1.09	3.64 ± 1.59	0.063
Sacral cord	1.13 ± 1.512	4.31 ±1.5	0.063

RABV = Rabies virus, SD = Standard deviation.

*p values were calculated using the Mann-Whitney U test. Underlines indicate statistical significance

**Fig 4:**
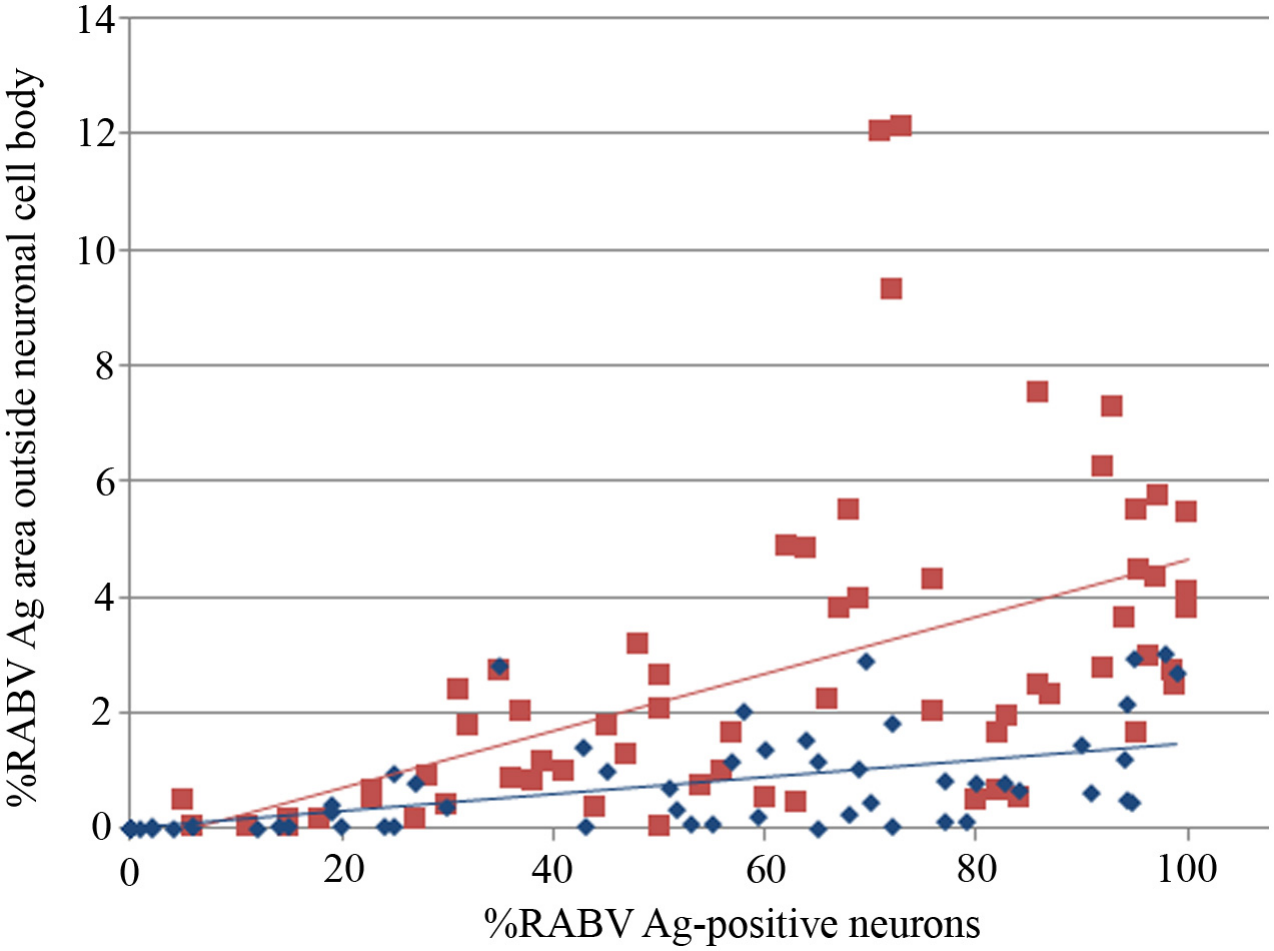
Graph of the percentage of areas in cellular processes positive for rabies antigen (Y axis) vs. the percentage of neuronal cell bodies positive for rabies antigen (X axis). Data points and linear regression line from dogs with furious rabies are shown in red and paralytic rabies in blue. RABV = Rabies virus, Ag = antigen.

The percentage of the antigen-positive area in cellular processes was compared to antigen parameters inside the neuronal cell body, using the data obtained from a previous study on the same set of animals [2] (Fig 4). Increasing percentage of positive neurons was strongly associated with greater antigen area beyond neuronal cell body in dogs with paralytic rabies (p<0.001, r = 0.6) and furious rabies (p<0.001, r = 0.51). From linear regression analysis, the equation generated for paralytic dogs was: Antigen area beyond neuronal cell body = 0.003 + 0.014 X percentage of positive neurons; and for furious dogs: Antigen area beyond neuronal cell body = 0.003 + 0.015 X percentage of positive neurons. The regression model also demonstrated that for a given percentage of positive neurons, the percentage of antigen area beyond neuronal cell body was significantly greater in furious compared to paralytic rabies (p<0.001).

**Fig 5:**
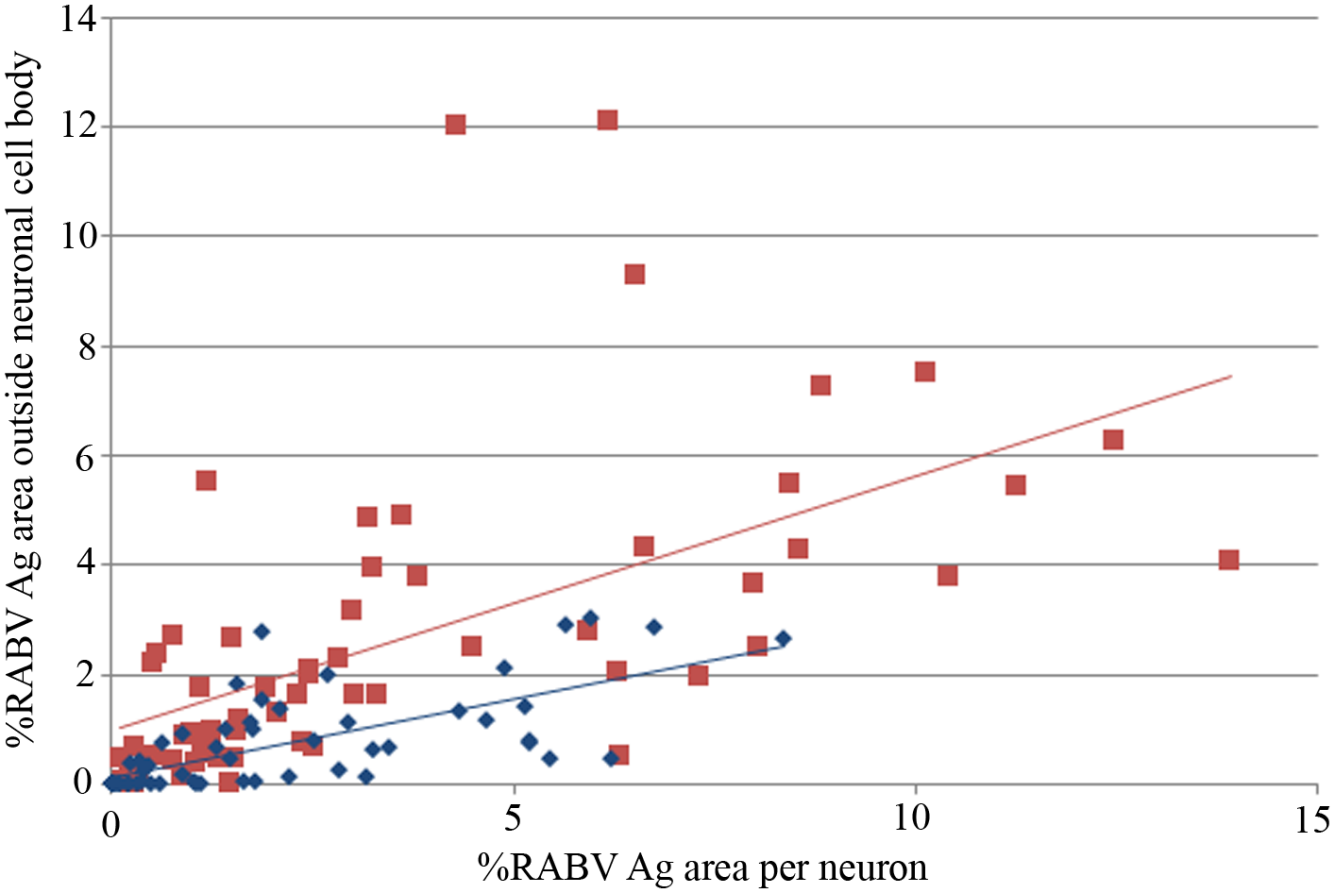
Graph of the area in cellular processes positive for rabies antigen (Y axis) vs. the antigen-positive area per neuron (X axis). Data points and linear regression line from dogs with furious rabies are shown in red and paralytic rabies in blue. RABV = Rabies virus, Ag = antigen.

Infected neurons were scored simply as positive or negative, regardless of the amount of antigen within the neuron. Hence, it was possible that reduced antigen in cell processes was simply the result of reduced antigen within the cell body. To investigate this, the areas positive for antigen in these two cell sites were compared (Fig 5). Similar to the percentage of infected neurons, antigen area per neuron also showed a strong correlation with the antigen area beyond neuronal cell body in dogs with paralytic rabies (p<0.001, r = 0.72) and furious rabies (p<0.001, r = 0.60). For both clinical subtypes, the amount of antigen beyond the neuronal cell body was always less than the amount within the cell body. Moreover, for the same amount of rabies antigen within the cell body, there was less rabies antigen within the cell processes in paralytic dogs compared to furious. From linear regression analysis, the equation generated for paralytic dogs was: Antigen area beyond neuronal cell body = 0.10 + 0.29 X antigen area within neuronal cell body; and for furious dogs: Antigen area beyond neuronal cell body = 0.98 + 0.46 X antigen area within neuronal cell body. The regression model also showed that, for a given antigen area within the neuronal cell body, the antigen area beyond neuronal cell body was significantly greater in furious compared to paralytic rabies (p<0.001). Thus, by both analyses, there was significantly less rabies antigen beyond neuronal cell body in dogs with the paralytic form, even when the degree of neuronal involvement was the same in both paralytic and furious dogs.

## Discussion

Considerable progress has been made in understanding the pathogenesis of the clinical diversity of rabies [3]. RABV enters the peripheral nervous system and undertakes long-distance transport, reaching at the cell soma and subsequently the CNS [12]. Initial uptake of virus begins at motor endplates or axons, followed by retrograde axonal transport to first order neurons, where viral transcription and replication occurs. Restricted first in the neuronal cell body, the virus is later transported intracellularly to dendrites. Transneuronal transfer occurs in retrograde direction, from neuronal cell bodies and dendrites to presynaptic terminals. From presynaptic terminals, virus particles are then transported back to the cell body of higher-order neurons, where the next transcription and replication cycle begins [13]. Using live cell imaging, it has been shown that RABV binding to p75 neurotrophin receptor (p75NTR) affects the axonal transport process by hijacking the neurotrophic factor endocytosis and retrograde transport machinery, resulting in a faster and more efficient way for RABV to reach the cell body [14].

Examination of subjects at the early stage of disease (as in our study) offers the opportunity to observe the patterns of infection in the two clinical forms. Moreover, detection of rabies antigen provides a visual representation of the spread of the virus. The results of the current study on antigen in cellular processes at different levels of the CNS extend the results from our previous study on antigen with neuronal cell bodies and viral RNA levels [2, 6]. In keeping with the retrograde spread of the rabies virus, caudal-rostral polarity of viral antigen distribution within neurons was observed in both furious and paralytic forms in order (from greatest to least): spinal cord, brainstem, cerebellum, midline structures (caudate, thalamus), hippocampus, and cerebrum [2, 3]. The distribution of viral antigen in dogs is generally the same as in the human cases (i.e., greater amount of viral antigen in the midline including brainstem, thalamus as compared to other supratentorial structures) [15]. The thalamus and brainstem were also found to have RABV antigen in all brain samples infected with rabies in a variety of animal species [16]. Virus antigen levels in cell processes beyond the neuronal cell body were always lower than neuronal cell bodies, regardless of clinical form, reflecting that transcription and translation occurs in the cell body, with later transport out to cell processes. Again, a caudal-rostral polarity of viral antigen distribution was generally observed within cell processes, consistent with the clinical findings that the trunk or most caudal portions of the CNS are usually infected first, providing more time for viral transport out to cell processes, than for more rostral structures. The antigen distribution results contrast with the RNA levels, which are highest in the cerebral midline structures and cerebrum, particularly in the furious form. This finding again reflects the ascending route of rabies infection. These higher CNS areas are infected last, and at the time of animal sacrifice, are the sites of most active viral replication, before formation of viral particles, hence low antigen detection in both cell bodies and processes by immunohistochemistry. In contrast, caudal levels, which are infected first, are past the stage of active replication and show low RNA levels with higher amounts of virus particles and higher antigen detection.

We cannot comment on the speed of infection since our study was at single time point at which infected animals showed early signs of disease. The time course for spread of rabies virus is remarkably similar in experimental models and in street strain-infected human patients [3]. Upon reaching first order neurons in the spinal cord, the virus takes 0.5 days to travel to the next higher order neuron and follows a similar pattern in a non-distance-dependent active transport mode regardless of the distance. The virus can stay silent within the CNS after reaching the first order neuron for at least three weeks. During that time, abnormalities can be demonstrated in the spinal cord and brain of patients even without any cerebral symptoms [17]. However, anterograde virus propagation from infected neurons to the periphery along sensory nerve starts after 48 hours in that infected neuron. This process is much slower, requiring a longer time to reach sites such as dorsal root ganglia, skin, hair follicles, and lymph nodes.

A significantly greater amount of viral antigen was observed beyond the neuronal cell body (within neuronal processes) in the furious subtype, as compared to the paralytic, in several CNS regions including almost all cerebral lobes. Our previous studies in animals and humans did not examine the hippocampal region thoroughly [5, 6]. Pyramidal neuron cell bodies in the hippocampal cornu ammonis were found to be more severely affected in the furious rabies [2]. A similar trend was observed in the present study for the cellular processes of the pyramidal neurons, as well as in the dentate fascia. Thus, several components of the hippocampus are more involved in furious dogs, in which limbic symptoms predominate.

The most interesting observation was the discrepancy in the degree of antigen content beyond the neuronal cell body between furious and paralytic rabies. While there was a general trend of increasing values for antigen in cell processes as the number of infected neurons increased, there was significantly more antigen in furious dogs than in paralytic dogs, comparing the same percentage of neuronal involvement. This was not simply the result of less antigen per neuron cell body in paralytic dogs. Comparing neuron cell bodies with the same amount of rabies antigen, there was still less antigen in cell processes in paralytic compared to furious dogs. This implies the transport of rabies from the cell body to cell processes is faster or more efficient in the furious form compared to the paralytic form. This may be one of the mechanisms by which the furious subtype achieves a more rapid viral colonization of the CNS, in addition to greater antigen levels within neurons and higher RNA levels that have been documented previously [2, 6].

The basis for the slower transport of virus to cellular processes in the paralytic form remains intriguing. One possibility is a genetic difference between the rabies viruses in the two clinical forms that influences intracellular transport of the virus. We have shown that virus isolated from the brainstem from furious and paralytic rabies-infected patients showed only minor nucleotide difference in the nucleocapsid (N), glycoprotein (G) and phosphoprotein (P) genes that did not translate into significant differences at the protein level [18]. However, this study was based on postmortem specimens sampling only one particular region of the brain. Other levels of the CNS need to be studied. It remains possible that particular viral genetic elements may influence the dynamics of spread and even the adaptation of viral mechanisms to evade the immune system [3]. Alternatively (or concurrently), this might be a host-related phenomenon, and could be related to the immune response and/or cytokines. We previously reported inflammation consisting mainly of T lymphocytes in the brainstem of dogs with paralytic rabies, and postulated this may impede viral propagation to cerebral hemispheres in this subtype [2]. Also, at the early stage of rabies infection in dogs, interleukin-1β and interferon-γ mRNAs were found exclusively in paralytic cases [6].

Since the neuropathological changes in rabies infection are very mild, neuronal dysfunction has been postulated as the basis for severe clinical disease [19]. In experimental models, pathogenic RABV has been shown to cause degeneration of neuronal processes (both dendrites and axons) by disrupting cytoskeletal integrity [19, 20]. Rabies virus phosphoprotein interacts with mitochondrial Complex I and induces mitochondrial dysfunction and oxidative stress, leading to axonal injury [19, 21]. Degeneration of the neuronal processes may have an impact on viral transport from cell body to cell processes. Although this possibility deserves further evaluation, rabies caused by street (wild-type) virus strains may not follow the same mechanisms as experimental models using laboratory rabies virus strain. For example, neuronal apoptosis is almost exclusively induced by laboratory rather than wild-type strains of RABV [22]. Therefore, even though there are factors that cannot be tightly controlled in the natural setting (amount of virus infected, stage of disease when the animals are terminated, etc.), studying the natural infection has advantages to understanding rabies infection. Further studies are needed to determine the mechanism that delays transportation of RABV within the neuronal cell body to the cell processes in paralytic as compared to furious rabies.
